# Alterations in Erythrocyte and Platelet Characteristics Are Poor Indicators of Metastasis in Dogs with Carcinoma or Sarcoma: A Preliminary Study

**DOI:** 10.3390/vetsci13050465

**Published:** 2026-05-11

**Authors:** Adriana A. Mulder, Amelia Goddard, Paolo Pazzi

**Affiliations:** 1 Department of Companion Animal Clinical Studies, Faculty of Veterinary Science, University of Pretoria, Pretoria 0110, South Africa; aletta.mulder@tah.co.za (A.A.M.); amelia.goddard@up.ac.za (A.G.); 2 Tygerberg Animal Hospital, 1 Kontiki Avenue, Glen Ive, Cape Town 7550, South Africa; 3 Department of Small Animal Clinical Sciences, College of Veterinary Medicine, University of Tennessee, Knoxville, TN 37996, USA

**Keywords:** canine, cancer, erythrocyte, platelet, morphology, inflammation

## Abstract

Cancer is a leading cause of death in humans and dogs. Several erythrocyte and platelet characteristics (indices and morphology) have shown promise as indicators of metastasis in humans. Similar studies have not been performed in dogs. This study evaluated erythrocyte and platelet characteristics measured on the Advia 2120i in 59 tumor-bearing dogs with carcinoma or sarcoma. Tumor-bearing dogs with and without intracavitary hemorrhage that underwent complete post-mortem and histopathology examinations were compared to healthy age-controlled dogs. Carcinoma- and sarcoma-bearing dogs without hemorrhage were compared. All tumor-bearing dogs without hemorrhage or metastasis were compared to those with metastasis, and characteristics were evaluated as indicators of metastasis. Tumor-bearing dogs without intracavitary hemorrhage (*n* = 49) had decreased hematocrit (*p* = 0.002) and reticulocyte hemoglobin content (*p* = 0.022), and increase in anisocytosis (*p* = 0.002), polychromasia (*p* = 0.002), macrocytosis (*p* = 0.032), codocytes (*p* = 0.022), absolute reticulocyte count (*p* = 0.035), platelet concentration (*p* = 0.002), plateletcrit (*p* = 0.022), and platelet volume distribution width (*p* = 0.022) compared to healthy dogs (*n* = 20). In tumor-bearing dogs with intracavitary hemorrhage (*n* = 10), additional significant differences were reflective of acute hemorrhage. No difference in characteristics between carcinoma- and sarcoma-bearing dogs without hemorrhage was identified. After correction for multiple comparisons, no differences in erythrocyte or platelet characteristics were identified between tumor-bearing dogs without intracavitary hemorrhage and metastasis and those without metastasis. Significant differences in characteristics exist between tumor-bearing dogs and healthy dogs. Based on the limited number of dogs in this preliminary study, no red blood cell or platelet characteristics were associated with metastatic disease in tumor-bearing dogs without hemorrhage.

## Simple Summary

Cancer is a common cause of death in both people and dogs. In humans, changes in red blood cells and platelets have been explored as possible indicators of cancer spread (metastasis), but this has not been well studied in dogs. In this study, we evaluated blood cell characteristics in dogs with cancer and compared them to those of healthy dogs. We found that dogs with tumors showed several changes in red blood cells and platelets, particularly in dogs without internal bleeding. However, these changes were similar between different tumor types and were not associated with whether the cancer had spread. Based on the limited number of cases, blood cell characteristics alone were not useful for identifying metastasis in dogs without hemorrhage.

## 1. Introduction

In humans, almost 20 million new cancer cases and an estimated 9.7 million deaths were reported by the global cancer observatory in 2022 [1]. Due to the shortage of public health services worldwide and the cost associated with the treatment of cancer in humans, an increasing number of studies have focused on finding indicators of metastatic disease for preliminary and cost-effective classification of patients. Similar studies are limited in dogs with cancer.

A complete blood count (CBC) is a minimally invasive test that forms part of the preliminary work-up when a patient is presented for diagnostic evaluation. Erythrocyte and platelet indices have been studied extensively as indicators of the presence of metastasis in humans with cancers, while their predictive potential for metastasis in dogs is largely unknown [2,3,4]. Indices or markers associated with metastasis in dogs include the expression of parathyroid hormone receptor 1(PTHR1) in canine osteosarcoma tissue; dogs with high immunostaining intensity for PTHR1 had significantly shortened survival [5]. Dogs with metastatic splenic hemangiosarcoma and perioperative thrombocytopenia were associated with a shorter overall survival time, and low concentrations of deltaNp63 expression in the urinary bladder were associated with vascular invasion, metastasis, and shortened survival in dogs with transitional cell carcinoma [6,7]. In humans diagnosed with cancer, changes in various erythrocytes and platelet indices were reported in patients with metastasis, and changes were also predictive of possible chemotherapy resistance [3,8,9]. In patients with oral squamous cell carcinoma, a red cell distribution width (RDW) ≥ 15% was associated with lymph node metastasis, platelet concentration (PLT) ≥ 400 × 10^9^/L was found to be an independent predictor of metastasis in colorectal cancer, mean platelet volume (MPV) was significantly higher in patients with metastatic colorectal cancer and an increase in platelet volume distribution width (PDW) was associated both with the depth of invasion and lymph nodes metastasis in patients with nasopharyngeal carcinoma [3,4,9,10]. Changes to these indices are attributed to various mechanisms, including blood loss from the tumor itself and cytokine production, including interleukin 6 (IL-6) and tumor necrosis factor-α (TNF-α), by tumors and associated inflammatory cells, leading to platelet activation and eventually malnutrition [2,3]. Interleukin 6 promotes the production of acute phase proteins, such as C-reactive protein (CRP), and serum amyloid A (SAA) in the liver, with both CRP and SAA used as biomarkers of an acute inflammatory response. Although typically increased in the presence of acute inflammation, an increase in CRP and SAA concentrations has also been reported in chronic disease, including cancer [11,12]. In addition to promoting the production of CRP and SAA, (IL) 6 also promotes the production of hepcidin with resultant decrease in the availability of iron [13]. In contrast to erythrocyte and platelet indices, the predictive value of abnormal erythrocyte morphology for the presence of metastasis is unknown in both humans and dogs with carcinoma and sarcoma.

Considering the morbidity, mortality, welfare and cost implications of cancer, studies aimed at establishing minimally invasive and cost-effective prognostic indicators in dogs, including parameters based on CBC characteristics that would benefit the patient, clinician, and owner. The primary objectives of this study were to (1) compare erythrocyte and platelet characteristics (indices and morphology) between tumor-bearing dogs and age-controlled healthy control dogs. Due to the effect of hemorrhage on erythrocyte and platelet characteristics, dogs with and without intracavitary hemorrhage were compared as two separate groups with healthy age-controlled dogs [14,15]; (2) compare characteristics between dogs with carcinoma and sarcoma without intracavitary hemorrhage; and (3) identify significant erythrocyte and platelet characteristic differences between tumor-bearing dogs without hemorrhage, with and without metastasis, that might act as indicators of metastasis. The secondary objective was to identify correlations between acute phase proteins, CRP and SAA, and altered erythrocyte and platelet indices in tumor-bearing dogs. We hypothesize that erythrocyte and platelet characteristics will be altered in tumor-bearing dogs when compared to healthy age-controlled dogs; there will be no significant difference in characteristics between the carcinoma and sarcoma tumor types, and some characteristics will be indicators for metastasis in tumor-bearing dogs without intracavitary hemorrhage. Additionally, we hypothesize that the degree of inflammation, based on changes in CRP and SAA, will correlate with alterations in erythrocyte and platelet characteristics in tumor-bearing dogs.

## 2. Material and Methods

### 2.1. Experimental Design

This cross-sectional study evaluated the erythrocyte and platelet indices, as well as erythrocyte and platelet morphology, on samples prospectively collected for a study in dogs diagnosed with carcinoma or sarcoma [16]. The cohort of study animals considered for inclusion was dogs that presented to Onderstepoort Veterinary Academic Hospital, University of Pretoria between December 2018 and September 2020 with an exclusive diagnosis of carcinoma or sarcoma, based on clinical and cytological examination. Dogs were not considered for inclusion if they had evidence of trauma or other non-tumor-related inflammatory conditions, or another tumor type identified during clinical examination. Only tumor-bearing dogs where owners elected for euthanasia were included, after owner consent was obtained. The decision by owners to elect euthanasia was influenced by several factors, including financial constraints and the owners’ perception of quality of life when confronted by a diagnosis of neoplasia. A CBC and post-mortem were performed on all tumor-bearing dogs, and carcinoma or sarcoma was confirmed on histopathological examination.

The cohort of healthy, age-controlled control animals considered for inclusion consisted of 20 staff, client, and student-owned dogs. With owner consent, dogs were included if they were older than 9 years of age, clinically healthy based on no evidence of illness in the preceding four weeks, normal clinical examination, abdominal ultrasound, and three-view thoracic radiographs. Dogs were excluded from the healthy unaffected group if there was evidence of tumors or inflammatory conditions, gross abnormalities on abdominal ultrasound or three-view thoracic radiographs. The study was approved by the Research Ethics Committee and Animal Ethics Committee of the University of Pretoria (REC185-22).

### 2.2. Experimental Procedure

After obtaining a history from the owner, a physical examination, fecal floatation, urinalysis, and CBC and blood smear evaluation were performed for all dogs. Post-mortem examination, including sample collection, was only performed in the tumor-bearing dogs. Blood samples were collected in a serum tube first and then an EDTA tube (Beckton Dickinson Vacutainer Systems, Wokingham, UK) via jugular venipuncture with a 21-gauge needle, using vacuum assistance. Blood was collected from tumor-bearing dogs less than one hour before euthanasia and from control dogs after being deemed healthy.

A CBC was performed on the EDTA sample within two hours of collection on the Advia 2120i (Siemens, Munich, Germany). The variables measured included, hemoglobin (HGB), red blood cell count (RBC), hematocrit (HCT), mean corpuscular volume (MCV), mean corpuscular hemoglobin concentration (MCHC), reticulocyte percentage (RET%), absolute reticulocyte count (ARC), reticulocyte hemoglobin content (CHr), reticulocyte mean cell volume (rMCV), platelet concentration (PLT), plateletcrit (PCT), mean platelet volume (MPV), platelet volume distribution width (PDW), mean platelet component concentration (MPC), platelet component distribution width (PCDW), mean platelet mass (MPM), platelet dry mass distribution width (PMDW) and platelet aggregation. A blood smear was evaluated to verify platelet concentration, exclude blood-borne parasites, and identify morphological changes in erythrocytes and platelets. Erythrocyte and platelet morphological changes evaluated included anisocytosis, polychromasia, hypochromasia, macrocytosis, microcytosis, schistocytosis, poikilocytosis, echinocytes, spherocytes, codocytes, acanthocytes, keratocytes, leptocytes, Howell-Jolly bodies and giant platelets. The assessment of morphological changes was standardized in this study using the defined subjective grading as set out by Weiss [17]. All blood smears were visually assessed by a trained technician and verified by a boarded clinical pathologist, where necessary, regardless of any Advia 2120i flags. Briefly, alterations in erythrocyte and platelet morphology were evaluated in a monolayer (that did not include the feathered edge of the blood smear) at 1000× magnification for changes in volume, shape, color and cellular inclusions. Subjective grading of morphological changes utilized a scale of 0 to 3 with 0 = none, 1 = mild, 2 = moderate and 3 = severe changes. The only exception was the rating of anisocytosis and polychromasia with a grading scale of 0 to 4, with 0 = none, 1 = mild, 2 = moderate, 3 = prominent and 4 = severe [10].

The serum sample was separated by centrifugation and stored at −80 °C. Acute phase proteins were measured as a batch on the Cobas Integra 400 plus analyzer (Roche, Basel, Switzerland), and included CRP (Gentian, Moss, Norway) and SAA (Eiken, Tokyo, Japan) [18,19].

### 2.3. Post-Mortem Procedure and Sample Collection

A full post-mortem examination was performed within ninety minutes of euthanasia. Samples from the primary tumor, regional lymph nodes (when identified), and any other area displaying macroscopic changes or suspected metastasis were sampled for histopathological analysis and fixed in 10% formalin. Additional organ sampling involved collecting multiple 1 cm^3^ sections from all parenchymatous organs, including sections of lung (left cranial (cranial and caudal part) and caudal lobe, right cranial, middle and caudal lobe each); heart (left ventricle, atrium, right ventricle and atrium); liver (left medial and lateral, and the right medial and lateral lobes); spleen (head, body and tail); kidney (renal medulla and cortex at the cranial, middle and caudal aspects of each kidney); adrenals (transverse slices through the center of each adrenal); pancreas (right and left limb and body); and cerebrum (transverse section across each cerebral hemisphere). Furthermore, each parenchymatous organ was systematically sectioned both sagittally and transversely into approximately 1 cm blocks to allow for visual inspection and manual palpation for gross abnormalities or metastasis. Tissue samples were embedded and underwent standard hematoxylin and eosin staining and were evaluated by a board-certified pathologist. Intracavitary hemorrhage was defined as the presence of free fluid within the peritoneal, pleural, or pericardial cavity, with a HCT comparable to that of the peripheral blood of the patient. Recorded observations encompassed the anatomical location of the primary tumor, the presence of either grossly visible ulceration or histopathological evidence of inflammatory changes or necrosis affecting the tumor itself or organs, and the identification of additional primary neoplasms (secondary or tertiary) discovered during post-mortem examination or through histopathological evaluation.

### 2.4. Statistical Analysis

Statistical analysis was performed using SPSS^®^ 27 software (IBM SPSS Inc. Armonk, New York, NY, USA). Normality of data was evaluated using the Shapiro–Wilk test and histograms. To specifically determine the effect of tumors on erythrocyte and platelet indices, and morphological changes, tumor-bearing dogs with macrothrombi were excluded from further analysis due to the significant effect of macrothrombi on the HCT and platelet concentration [20]. For continuous variables, including erythrocyte and platelet indices, the Mann–Whitney U test was used to compare tumor-bearing dogs without intracavitary hemorrhage and controls, tumor-bearing dogs with intracavitary hemorrhage and controls, and finally, after exclusion of dogs with intracavitary hemorrhage, between tumor-bearing dogs with and without metastasis. For categorical variables, including breed, age, sex and erythrocyte and platelet morphology, the Fisher–Freeman–Halton exact test was used to compare the same groups previously described. Correlation between the acute phase proteins, CRP and SAA, and alterations in erythrocyte and platelet indices in tumor-bearing dogs without hemorrhage were evaluated with Spearman’s rank correlations. Correlation between CRP and SAA, and alterations in erythrocytes and platelet indices in tumor-bearing dogs with hemorrhage, were not evaluated due to the small sample size. To account for multiple comparisons, the Benjamini–Hochberg false discovery rate correction (q < 0.05) was applied to any data set in which a *p* < 0.05 was identified. Values reported as *p* < 0.001 or *p* < 0.01 by the statistics software were assumed to be *p* = 0.001 or *p* = 0.01 for the Benjamini–Hochberg correction, respectively. Data was presented as median and interquartile range (IQR). Significance was set at *p* < 0.05.

## 3. Results

### 3.1. Study Population

The study population consisted of 62 dogs that had a histopathological diagnosis of either sarcoma or carcinoma on necropsy. Three dogs were excluded from analysis due to the presence of macrothrombi. The remaining tumor-bearing group consisted of 59 dogs and 22 breeds (Table A1). Intracavitary hemorrhage was absent in 49 of the tumor-bearing dogs. The 20 healthy, unaffected dogs were above the age of 9 years and were represented by nine breeds. When breed categories, age, and sex were compared, only neutered status was significantly higher in the control group than in tumor-bearing dogs (*p* = 0.001).

**Figure 1:**
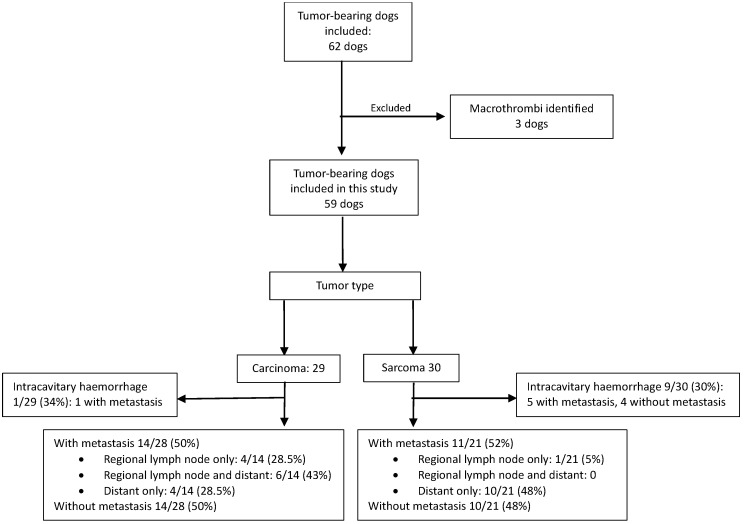
Flow diagram of case selection and distribution of tumor type, intracavitary hemorrhage, and metastatic status in tumor-bearing dogs.

Sarcoma was diagnosed in 30 dogs, with metastasis present in 16/30 (53%) dogs. Carcinoma was diagnosed in 29 dogs, with metastasis in 15/29 (52%). When dogs with intracavitary hemorrhage were excluded from the metastasis subgroup analysis, metastasis was present in 11/21 (52%) dogs with sarcoma and 14/28 (50%) dogs with carcinoma. Table 1 and Figure 1 provide details regarding the primary tumor and site/s of metastasis, and details secondary primary tumors that were identified in 10/29 (34%) dogs with carcinoma and 14/30 (47%) dogs with sarcoma.

### 3.2. Histopathological Evidence of Inflammation Associated with Tumors and Inflammation Unrelated to Tumors

Evidence of macroscopic ulceration or histopathological findings indicative of inflammation or necrosis involving the primary tumor or other organs was identified in 27 of 31 (87%) dogs with metastatic disease and in 21 of 28 (75%) dogs without metastasis (Table A2). Inflammation unrelated to tumors was present in 15 dogs in the study population.

### 3.3. Erythrocyte and Platelet Characteristics Compared Between Tumor-Bearing Dogs Without Intracavitary Hemorrhage (n = 49) and Healthy Unaffected Dogs (n = 20)

A significant increase was seen for RET%, ARC, PLT, PDW, PCT, anisocytosis and polychromasia in tumor-bearing dogs without intracavitary hemorrhage compared to healthy unaffected dogs, while significant decreases were seen for HGB, RBC, HCT and CHr (Table 2). Both CRP and SAA were significantly increased in tumor-bearing dogs when compared to healthy, unaffected dogs. Both CRP and SAA were moderately to strongly negatively correlated with HGB, RBC, HCT and CHr (Table 2).

### 3.4. Erythrocyte and Platelet Characteristics Compared Between Tumor-Bearing Dogs with Intracavitary Hemorrhage (n = 10) and Healthy Unaffected Dogs (n = 20)

A significant increase was seen for RDW, RET%, ARC, MPV, PDW, MPM, PMDW, anisocytosis, hypochromasia, polychromasia, codocytes, acanthocytes, keratocytes, leptocytes, macrocytosis, Howell-Jolly bodies, schistocytes and giant platelets, and a significant decrease was seen for RBC, HCT, MCHC, PLT and PCT in tumor-bearing dogs with intracavitary hemorrhage compared to healthy unaffected dogs (Table 3). Both CRP and SAA were also significantly elevated in tumor-bearing dogs when compared to healthy, unaffected dogs.

### 3.5. Erythrocyte and Platelet Characteristics Compared in Tumor-Bearing Dogs, Without Intracavitary Hemorrhage, with Sarcoma (n = 21) and with Carcinoma (n = 28)

There were no significant differences found when comparing the characteristics of tumor-bearing dogs with sarcoma to the characteristics of tumor-bearing dogs with carcinoma (Table 4).

### 3.6. Erythrocyte and Platelet Characteristics in Tumor-Bearing Dogs Without Intracavitary Hemorrhage, with (n = 25) and Without (n = 24) Metastasis

There were no significant changes in characteristics found after correction for multiple comparisons when comparing tumor-bearing dogs with metastasis to those without. (Table 5). The CRP and SAA concentrations were not significantly different between the groups and only SAA was strongly negatively correlated with CHr in tumor-bearing dogs without metastasis.

## 4. Discussion

Our preliminary study investigated erythrocyte and platelet indices and morphology in dogs diagnosed with carcinoma or sarcoma. Compared to healthy age-controlled dogs, tumor-bearing dogs without intracavitary hemorrhage showed a decrease in the RBC, with normal erythrocyte morphology and increased regeneration. Tumor-bearing dogs with intracavitary hemorrhage showed a significantly regenerative, based on ARC, normocytic hypochromic anemia, compared to the healthy age-controlled dogs. The PLT was significantly higher, with significant variation in MPV in tumor-bearing dogs without intracavitary hemorrhage compared to controls. In contrast, the PLT were significantly lower and MPV significantly higher in tumor-bearing dogs with intracavitary hemorrhage when compared to controls. Based on acute phase proteins, CRP and SAA, inflammation was found to be significantly increased in tumor-bearing dogs. The increased inflammation was correlated with decreased HGB, RBC, HCT, and CHr. No significant differences in erythrocyte or platelet characteristics were identified between carcinoma- and sarcoma-bearing dogs. After correction for multiple comparisons, no erythrocyte or platelet characteristics were significantly different between dogs with metastasis, compared to the tumor-bearing dogs without metastasis, in this cohort of dogs.

A decrease in HGB, RBC, and HCT was previously reported in dogs with histiocytic sarcoma, hematological cancers, mast cell tumors, and hemangiosarcoma [21]. In our study, tumor-bearing dogs had a significantly decreased HGB, RBC, and HCT compared to healthy age-controlled dogs, which was more severe in those dogs with intracavitary hemorrhage. Etiologies of anemia in patients with cancer include, but are not limited to, acute or chronic hemorrhage of the tumor itself, myelophthisis, microangiopathic hemolytic anemia, malnutrition, and anemia of chronic inflammation [21]. In both humans and dogs with cancer, the mechanism for chronic inflammation is multifactorial but includes the expression of proinflammatory cytokines, such as interleukin 1β, IL-6 and tumor necrosis factor-α (TNF-α) by stromal, or tumor cells themselves, macrophages and monocytes [22,23]. The increase in IL-6 is proposed to promote the production of hepcidin. Hepcidin degrades ferroportin, a transmembrane protein that facilitates the export of cellular iron and promotes the uptake of iron into macrophages. Sequestered iron may be less available for incorporation into the HGB molecule and may contribute to decreased erythropoiesis [13]. In addition to promoting the production of hepcidin, IL-6, together with IL-1, IL-2 and TNF-α, also promotes the production of the acute phase proteins CRP and SAA [11,24]. In our study, these biomarkers of inflammation were increased, and although cytokine concentrations were not directly measured, both CRP and SAA had a moderate negative correlation with HGB, RBC, and HCT. An increase in CRP and SAA concentrations is typically found with acute inflammation, but has also been reported in chronic disease, including cancer [12,16,25]. This is evident in our study, where inflammation was present in 80% of tumor-bearing dogs, not only in association with tumors, but also secondary to comorbidities. A diagnosis of cancer was established by histopathologic evaluation of multiple organs at necropsy, and concomitant pathology was therefore likely detected more frequently than would be expected in a purely clinical study. Interpretation is complicated by the fact that inflammation is an integral component of the tumor microenvironment, arising from interactions between neoplastic cells, necrosis and infiltrating immune cells. Distinguishing neoplasia-associated from non-neoplastic inflammation is inherently challenging and would require comparison with an age-matched inflammatory cohort, which is difficult to standardize. Therefore, chronic inflammation secondary to tumors and concurrent comorbidities, with potential iron sequestration, represents a plausible mechanism for the significant decreases in HGB, RBC, and HCT in dogs with carcinoma and sarcoma when compared to healthy dogs in this study.

Iron-restricted anemia, secondary to chronic inflammation, is typically associated with an inappropriate regenerative response [26]. However, in our study, a significant increase in RET% and ARC was seen in tumor-bearing dogs without hemorrhage compared to healthy dogs. Although the increase in RET% and ARC is significant between groups, the median of RET% is only marginally above the published normal reference interval of 0.14–1.48%, while the ARC is below the reference of <95 × 10^9^/L as provided by the internal reference laboratory [27]. Although many tumor-bearing dogs were not considered to be anemic clinically, their median HGB, RBC, and HCT were significantly lower compared to the controls, and the severity of the decrease was associated with increased inflammation, based on CRP and SAA. The relative increase in reticulocytes could therefore be seen as an appropriate response to the relative decrease in HGB, RBC and HCT, hypothesized to be related to increased inflammation and iron sequestration, but a definitive etiology for this increase remains elusive. As ARC is not significantly correlated with CRP or SAA, the increase in reticulocytes could be related to processes unrelated to inflammation.

The CHr is seen as an objective estimation of the availability of iron, as it is the quantification of the amount of hemoglobin in reticulocytes. The CHr is therefore considered to be a measure of both the ability of the bone marrow to produce hemoglobin and the availability of iron to be utilized in erythropoiesis in humans with cancer [28]. A decreased CHr has previously been reported to be significantly correlated with an increase in CRP, and similarly, our study found a decreased CHr that showed a moderate negative correlation with both CRP and SAA [29]. This correlation in CHr is consistent with the possibility that decreased iron availability, secondary to inflammation and subsequent sequestration, is one of the mechanisms for the decrease in HGB, RBC and HCT seen in this study. However, the median CHr in tumor-bearing dogs without hemorrhage (24.7 pg), although significantly decreased compared to healthy unaffected dogs, is still above the reported lower limit of the CHr reference interval. With the lower limit reported as 22.3 pg to 24.5 pg by various studies, the severity of potential iron sequestration appears mild, but measurable [30,31]. The relatively mild anemia, with appropriate regeneration, in tumor-bearing dogs without intracavitary hemorrhage is subjectively supported by the significant increase in anisocytosis, polychromasia, macrocytosis and codocytes.

In the tumor-bearing dogs with intracavitary hemorrhage, several significant changes in erythrocyte indices and morphology were identified compared to healthy, unaffected dogs, some of which are associated with acute hemorrhage. Moderate anemia, secondary to acute hemorrhage, is objectively represented by a significantly decreased HGB, RBC and HCT. Anemia gives rise to hypoxia, and the resultant reticulocytosis and significantly increased ARC are a normal physiological response to the hypoxia [32]. The significantly increased RDW and anisocytosis in tumor-bearing dogs with intracavitary hemorrhage can also be explained by the significant increase in reticulocytes. The presence of polychromasia, Howell-Jolly bodies, and macrocytosis is a subjective description consistent with the presence of reticulocytosis. A significant increase in acanthocytes was also seen in the tumor-bearing dogs with intracavitary hemorrhage. Several mechanisms have been proposed for the formation of acanthocytes in dogs with cancer, including an imbalance between phospholipid and cholesterol in the lipid bilayer of the erythrocyte and damage to the erythrocyte due to microangiopathy [33]. Microangiopathy is often present in hemangiosarcomas due to fibrin accumulation in the vasculature and the formation of abnormal vessels in the tumor [34]. The presence of acanthocytes in dogs with hemangiosarcoma has been the subject of several previous studies, and although not pathognomonic for hemangiosarcoma, it is commonly present. Two studies reported acanthocytes to be present in half the dogs presented with hemangiosarcoma, and one study reported acanthocytes in all the dogs with hemangiosarcoma [33,35,36]. In our study population, nine of the ten dogs with intracavitary hemorrhage were diagnosed with hemangiosarcoma, and this could explain the increase in acanthocytes seen. Microangiopathic damage to erythrocytes, secondary to tumors, is also associated with the formation of keratocytes and schistocytes, which were both significantly increased in this group. When this mechanism is the cause of increased keratocytes, it is often associated with an increase in schistocytes, as seen in our study.

Compared to healthy, unaffected dogs, significantly increased PLT and PCT were identified in tumor-bearing dogs without hemorrhage. Increased PLT in humans and dogs with neoplasia is multifactorial and may, in part, occur due to increased production of proinflammatory cytokines, such as IL-6, by the tumor cells and tumor-associated macrophages, and through any inflammation or necrosis of the tumor, as noted in the majority of dogs in our study [37]. Interleukin 6 promotes the production of thrombopoietin by the liver with subsequent stimulation of the production and differentiation of megakaryocytes [38]. Increased PLT has been described in the presence of iron deficiency anemia through preferential formation of megakaryocyte progenitors, from the combined megakaryocytic/erythroid progenitor, resulting in thrombocytosis [39]. Therefore, iron deficiency anemia could play a role in the increased PLT in our study; however, total iron was not measured in our study, and the CHr would be expected to be lower for clinically significant iron deficiency to be present [40]. Additionally, reactive thrombocytosis due to the presence of chronic hemorrhage may also result in an increase in PLT [14]. The increased PCT in our study, the product of PLT and MPV, is more likely due to the noted increase in PLT, as the MPV did not differ significantly compared to the unaffected group. The PDW was significantly increased in tumor-bearing dogs without hemorrhage. PDW is an indicator of the variability of MPV. Mean platelet volume is the measure of the average size of circulating platelets and is influenced by the maturity and activation of platelets [41]. In dogs with cancer, platelets may be activated after exposure to sub-endothelial collagen of damaged vascular endothelium, as well as by tumor cells. Tumor cells can indirectly cause platelet activation through increased expression of tissue factor by endothelial cells, thereby changing the surface of the endothelial cells, making it more suitable for adhesion of platelets [42]. Upon activation, platelets change shape to become more orb-shaped and develop pseudopodia of different sizes, leading to platelets with larger volume. The mRNA present in large platelets is preferentially associated with the hemostatic process when compared to small platelets, leading to faster aggregation by large platelets and increased consumption [41]. Therefore, with no increase in MPV, the increase in PDW could be a result of the presence of both small, resting platelets and large, activated platelets, leading to an MPV within the normal reference interval.

In tumor-bearing dogs with intracavitary hemorrhage, a significant thrombocytopenia was present compared to healthy, unaffected dogs and was most likely attributed to consumption of platelets due to acute hemorrhage. The significantly decreased PCT is due to the presence of significant thrombocytopenia, while the increase in MPV most likely represented activated or immature platelets. Platelet volume is influenced by cytokines such as IL-6, reportedly due to early division of the megakaryocyte, during megakaryopoiesis and thrombopoiesis, but hemorrhage will result in platelet activation and increased platelet production with release of larger immature platelets from the bone marrow [41,43]. It has been described that giant platelets are also formed via pathways that are independent of thrombopoietin when there is an increase in platelet consumption [41]. These alternative pathways include the division of megakaryocytes under the control of interleukin 1α during states of acute platelet demand. The platelets produced under the control of interleukin 1α are larger than platelets produced under the control of thrombopoietin [44].

Due to the inclusion of various carcinomas and sarcomas, tumor heterogeneity as a result of hemorrhage, tumor type, inflammatory response and necrosis, iron metabolism and platelet activation may have affected the erythrocyte and platelet characteristics evaluated in this study. To control for the effect of recent hemorrhage, dogs with intracavitary hemorrhage at presentation, of which 80% were hemangiosarcoma, were evaluated as a separate group, thereby improving the homogeneity of the study population [45,46]. The remaining study population had no evidence of intracavitary hemorrhage, and soft tissue sarcoma, osteosarcoma, mammary carcinoma and cutaneous squamous cell carcinoma are associated with a low risk of hemorrhage [47,48,49,50].

No significant differences in erythrocyte and platelet characteristics were identified between carcinomas and sarcomas, suggesting that, at the level of tumor class, heterogeneity had a limited effect on the evaluated variables, although differences between individual tumor types cannot be excluded. Inflammation and necrosis are recognized features across many of the tumor types included in this study, including hemangiosarcoma, osteosarcoma [51,52], soft tissue sarcoma [49,53], mammary carcinoma [54,55,56], pulmonary carcinoma, urothelial carcinoma [57,58], and hepatocellular carcinoma [45,59,60,61,62], and are incorporated into histologic grading and prognostic scoring systems for several of these tumors. However, their severity varies both within and between tumor types. Similar alterations in iron metabolism and platelet dynamics have been reported across multiple tumor types, often in association with inflammation or chronic blood loss, but remain incompletely characterized at the level of individual neoplasms [34,60,63,64,65,66,67,68,69,70,71,72]. While biological heterogeneity cannot be fully eliminated, the exclusion of dogs with intracavitary hemorrhage, lack of observed differences between major tumor classes and the presence of shared pathological processes across tumors suggest that its impact on the primary outcomes of this study is likely limited.

The only characteristics that showed a significant difference between tumor-bearing dogs with and without metastasis were a mildly decreased MCV and giant platelets in dogs with metastasis. However, after false discovery rate correction, no variables remained significant. It must also be noted that the MCV for both tumor-bearing dogs with and without metastasis is still within accepted normal reference intervals of 62.7–74.56 fL, which is supported by the absence of microcytosis on the blood smear. This significant overlap in MCV values further decreases the clinical use of this finding.

The study included some limitations. Strict exclusion criteria limited the size of our population of tumor-bearing dogs, and small group sizes with correction for multiple comparisons increased the likelihood of a type II error; therefore, the results should be considered exploratory, and larger studies are required. The study design does not allow for the determination of whether observed changes are causal or consequential, and longitudinal studies are needed to clarify these effects. Ideally, individual tumor types should have been evaluated separately; however, doing so would have resulted in subgroup sample sizes too small to support meaningful statistical inference. The absence of significant differences between major tumor classes does not preclude more subtle, tumor-specific effects that may not have been detected in this cohort. Inclusion was limited to euthanized dogs, which may have introduced selection bias and reduced generalizability to the broader population of tumor-bearing dogs, particularly those with earlier-stage disease; however, euthanasia in this cohort was not exclusively associated with advanced disease severity, as some dogs were euthanized because surgery or further treatment was declined, despite the lack of advanced disease, for financial or other reasons. Metastasis was evaluated as a dichotomous variable, as consistent and comprehensive quantification of metastatic burden and distribution was not feasible, limiting assessment of their potential influence on hematologic parameters. Finally, the control population was limited by the availability of healthy older dogs, the timeframe for collection, and financial constraints. Based on the available group sizes (20 controls and 49 tumor-bearing dogs), the study would be expected to have approximately 80% power to detect only moderate-to-large between-group differences (standardized effect size approximately 0.75), whereas smaller differences may have gone undetected. Accordingly, the control group was considered sufficient for the exploratory comparison for this study, but not for excluding more subtle hematologic differences. Finally, previous studies have reported neutered dogs, irrespective of sex, had significantly higher HGB, MCHC and PLT and significantly lower PMDW than intact dogs [73,74]. The study population consisted of approximately 50% intact individuals compared to the control population, which consisted of 10% intact individuals. Of these variables, a significant difference between tumor-bearing dogs and the age-controlled dogs was only identified in HGB and PLT, and PLT were significantly lower in age-controlled dogs, in contrast to what would be expected if neuter status was playing a role. Despite this, it is not implausible that the presence of more intact individuals in the study population may have had an influence on these variables.

## 5. Conclusions

In this preliminary study, several erythrocyte and platelet characteristics showed significant differences between tumor-bearing dogs and a normal age-controlled population of dogs, and should alert the clinician to the possible presence of carcinoma or sarcoma, allowing focused diagnostics and preemptive treatment. Many erythrocyte indices in tumor-bearing dogs showed moderate correlations with inflammatory acute phase proteins CRP and SAA. Based on the mixed cohort of tumor-bearing dogs with often advanced disease in this study, erythrocyte and platelet characteristics do not appear to be useful for the prediction of metastasis. Future studies, with larger populations, should focus on a specific tumor to identify potential changes in tumor characteristics associated with metastasis.

## Figures and Tables

**Table 1:** Primary tumors of dogs with carcinoma or sarcoma and the site(s) of metastasis of the primary tumor.

Primary Tumor	Metastatic Site of Primary Tumor
Sarcoma (30)	
Subcutaneous soft tissue sarcoma	Lung
Subcutaneous soft tissue sarcoma	N/A
Subcutaneous soft tissue sarcoma	N/A
Splenic hemangiosarcoma	Right atrium, liver
Splenic hemangiosarcoma *	Lymph node, liver, kidney, omentum
Splenic hemangiosarcoma *	N/A
Right atrial hemangiosarcoma *	Spleen, lungs
Right atrial hemangiosarcoma *	N/A
Splenic & right atrial hemangiosarcoma *	Liver, omentum
Splenic & right atrial hemangiosarcoma *	N/A
Hepatic hemangiosarcoma *	Omentum
Hepatic hemangiosarcoma *	Diaphragm, lungs
Cutaneous hemangiosarcoma	N/A
Cutaneous hemangiosarcoma	N/A
Subcutaneous hemangiosarcoma	Lungs
Muscle hemangiosarcoma	Liver, right atrium, lungs
Mammary osteosarcoma	Lymph node
Mammary osteosarcoma	Lungs
Oesophageal osteosarcoma	Aorta, liver, lungs
Oesophageal osteosarcoma	Kidney, liver, lung, caudal vena cava
Rib chondroblastic osteosarcoma	Lung
Maxillary osteosarcoma	N/A
Mandibular osteosarcoma	N/A
Mixed peri-orbital anaplastic sarcoma	Liver, pancreas, omentum, cardiac muscle, dermis/subcutis & muscle
Splenic stromal sarcoma	Liver
Muscle soft tissue sarcoma	N/A
Mixed mammary sarcoma	N/A
Hepatic spindle cell sarcoma *	N/A
Cutaneous soft tissue sarcoma	N/A
Mandibular soft tissue sarcoma	N/A
**Carcinoma (29)**	
Complex mammary carcinoma	Lymph node
Complex mammary carcinoma	Lymph node
Complex mammary carcinoma	N/A
Complex mammary carcinoma	N/A
Simple tubular mammary carcinoma	Lymph node
Simple tubular mammary carcinoma	Lungs
Simple tubular mammary carcinoma	N/A
Mixed mammary carcinoma	Lymph node
Mixed mammary carcinoma	Lymph node, lungs
Anaplastic mammary carcinoma	Lymph node, lungs
Simple solid mammary carcinoma	N/A
Simple tubulopapillary mammary carcinoma	N/A
Mammary ductal carcinoma	N/A
Mixed type carcinoma & simple tubulopapillary mammary carcinoma	N/A
Spindle cell mammary carcinoma	N/A
Cutaneous squamous cell carcinoma	N/A
Cutaneous squamous cell carcinoma	N/A
Hepatocellular carcinoma *	Liver
Hepatocellular carcinoma	N/A
Apocrine gland adenocarcinoma (anal sac)	Lymph node, spleen, adrenal gland, lungs
Carcinoma (papillary & solid)—source unknown	Lungs
Tubular mesothelioma	Lungs
Anaplastic cholangiocellular carcinoma	Lymph node, liver, peri-pancreatic connective tissue, bronchial lnn., lungs
Thyroid microfollicular carcinoma	Rib
Urothelial carcinoma	Lymph node, rib, gastric and tracheobronchial lymph node
Pulmonary carcinomatosis	Lymph node, Adrenal gland, liver, kidney
Pulmonary carcinoma (tubulopapillary type)	N/A
Sinonasal transitional carcinoma	N/A
Solid scirrhous prostatic carcinoma	N/A

* Indicates intracavitary hemorrhage at post-mortem.

**Table 2:** Erythrocyte and platelet variables of tumor-bearing dogs (without intracavitary hemorrhage) compared to healthy age-controlled unaffected dogs, as well as correlations of CRP and SAA to erythrocyte and platelet variables.

Variable	Tumor-Bearing Dogs Without Intracavitary Hemorrhage * (*n* = 49)	Healthy Unaffected Dogs * (*n* = 20)	*p* Value ^†^	BH Corrected *p* Value	CRP r_s_ to Variable (*p* Value; BH Corrected *p* Value)	SAA r_s_ to Variable (*p* Value; BH Corrected *p* Value)
**Complete blood count**						
**Erythrocytes**						
Hemoglobin (g/L)	132 (100, 148)	175 (167, 183)	**<0.001**	**0.003**	−0.45 **(0.001; 0.009)**	−0.42 **(0.003; 0.025)**
Red blood cell count (×10^12^/L)	5.73 (4.74, 6.75)	7.93 (7.16, 8.49)	**<0.001**	**0.003**	−0.41 **(0.003; 0.012)**	−0.38 **(0.007; 0.030)**
Hematocrit (L/L)	0.39 (0.31, 0.44)	0.53 (0.50, 0.55)	**<0.001**	**0.003**	−0.44 **(0.002; 0.011)**	−0.40 **(0.005; 0.028)**
Mean cell volume (fL)	67.85 (65.8, 70.1)	68.6 (66.2, 71.8)	0.40	0.43	−0.11 (0.46; 0.49)	−0.09 (0.53; 0.56)
Mean cell hemoglobin concentration (g/dL)	33.0 (32.6, 33.7)	33.4 (32.8, 33.5)	0.32	0.38	-	-
Red cell distribution width (fL)	13.8 (13.1, 15.4)	13.6 (13.05, 13.85)	0.151	0.22	0.27 (0.06; 0.13)	0.30 (**0.039**; 0.11)
Reticulocyte percentage (%)	1.8 (1.1, 2.8)	0.95 (0.63, 1.08)	**<0.001**	**0.003**	0.3 (**0.038**; 0.09)	0.31 (**0.033**; 0.11)
Absolute reticulocyte count (×10^9^/L)	94.8 (66.9, 159)	67.6 (48.4, 84.6)	**0.02**	**0.04**	0.15 (0.31; 0.40)	0.17 (0.25; 0.43)
Reticulocyte hemoglobin content (pg/cell)	24.7 (23.1, 25.4)	26.0 (24.8, 26.5)	**0.01**	**0.024**	−0.42 **(0.001; 0.009)**	−0.48 **(<0.001; 0.017)**
Reticulocyte mean cell volume (fL)	87.15 (83.1, 90.1)	88 (85.5, 92.3)	0.34	0.38	−0.25 (0.09; 0.17)	−0.24 (0.10; 0.19)
**Erythrocyte morphology**						
Anisocytosis	2 (1, 2)	1 (1, 1)	**0.001**	**0.003**	-	-
Polychromasia	1 (0, 1)	0 (0, 0)	**0.011**	**0.024**	-	-
Codocytes	0 (0, 1)	0 (0, 0)	**0.029**	0.051	-	-
Macrocytosis	1 (0, 1)	0 (0, 1)	0.052	0.08	-	-
**Platelets**						
Platelet concentration (×10^9^/L)	400 (288, 586)	268 (228, 299)	**<0.001**	**0.003**	0.09 (0.56; 0.56)	0.09 (0.55; 0.55)
Mean platelet volume (fL)	12.3 (10.5, 13)	10.9 (10.4, 12.1)	0.22	0.30	0.32 (**0.026**; 0.07)	0.24 (0.09, 0.19)
Platelet volume distribution width (%)	64.0 (60.5, 67.9)	57.2 (52.2, 62.3)	**<0.01**	**0.024**	0.27 (0.12; 0.20)	0.13 (0.38; 0.50)
Plateletcrit (%)	0.47 (0.33, 0.61)	0.3 (0.25, 0.37)	**<0.01**	**0.024**	0.17 (0.23; 0.36)	0.15 (0.29; 0.43)
Mean platelet component (g/dL)	21.2 (19.9, 22.3)	21.1 (20.1, 21.6)	0.86	0.89	−0.13 (0.35; 0.40)	−0.15 (0.31; 0.44)
Platelet component distribution width (g/dL)	6.5 (5.5, 7.2)	6 (4.4, 6.85)	0.055	0.08	−0.15 (0.31; 0.40)	−0.11 (0.46; 0.53)
Mean platelet mass (pg)	2.12 (1.97, 2.35)	2.12 (2.03, 2.315)	0.91	0.91	0.16 (0.28; 0.40)	0.13 (0.39; 0.49)
Platelet dry mass distribution width (pg)	0.88 (0.79, 1.0)	0.8 (0.76, 0.87)	0.052	0.08	0.34 (**0.018**; 0.06)	0.29 (**0.046**; 0.11)
Platelet Aggregation	0 (0, 1)	1 (0, 2)	0.28	0.35	-	-
**Acute phase proteins**						
C-reactive protein (mg/L)	65.0 (42.8, 142)	<10 (<10, <10)	**<0.001**	**0.003**	-	-
Serum amyloid A (mg/L)	25.9 (9.67, 122)	<2 (<2, <2)	**<0.001**	**0.003**	-	-

* Presented as median (interquartile range). Spearman’s coefficient is denoted by r_s._ ^†^ Based on Mann–Whitney U tests, with significance set as *p* < 0.05. The *p*-value for morphology based on Fisher–Freeman–Halton exact test; the *p*-value for Howell-Jolly bodies, poikilocytosis, platelet aggregation and giant platelets was not significant, and a *p*-value could not be calculated for the following morphological variables as the median and IQRs were zero: echinocytes, spherocytes, acanthocytes, hypochromasia, keratocytes, leptocytes, microcytosis and schistocytes, etc. Correlation is seen as significant at the 0.05 level (2-tailed). BH: Benjamini–Hochberg.

**Table 3:** Erythrocyte and platelet characteristics of tumor-bearing dogs (with intracavitary hemorrhage) compared to the variables of an age-controlled healthy population of dogs.

Variable	Tumor-Bearing Dogs with Intracavitary Hemorrhage * (*n* = 10)	Healthy Unaffected Dogs * (*n* = 20)	*p* Value ^†^	BH Corrected *p* Value
**Complete blood count**				
**Erythrocytes**				
Hemoglobin (g/L)	61.5 (49, 104)	175 (169, 183)	**<0.001**	**0.002**
Red blood cell count (×10^12^/L)	2.58 (2.29, 4.29)	7.93 (7.16, 8.49)	**<0.001**	0.002
Hematocrit (%)	0.20 (0.17, 0.31)	0.53 (0.50, 0.55)	**<0.01**	**0.015**
Mean cell volume (fL)	73.3 (68.1, 77.4)	68.6 (66.2, 71.8)	0.14	0.16
Mean cell hemoglobin concentration (g/dL)	32.4 (29.1, 33.1)	33.4 (32.8, 33.5)	**0.036**	**0.044**
Red cell distribution width (fL)	17.4 (15.5, 19.4)	13.6 (13.0, 13.8)	**<0.001**	**0.002**
Reticulocyte percentage (%)	10.6 (7.4, 21.1)	0.95 (0.65, 1.08)	**<0.001**	**0.002**
Absolute reticulocyte count (×10^9^/L)	289 (205, 447)	67.6 (48.4, 84.6)	**<0.001**	**0.002**
Reticulocyte hemoglobin content (pg/cell)	24.6 (23.7, 26.1)	26 (24.8, 26.5)	0.33	0.35
Reticulocyte mean cell volume (fL)	93.0 (86.3, 101)	88 (85.5, 92.3)	0.09	0.11
**Erythrocyte morphology**				
Anisocytosis	2.5 (2, 3)	1 (1, 1)	**<0.001**	**0.002**
Polychromasia	1 (1, 2)	0 (0, 0)	**<0.001**	**0.002**
Codocytes	0 (0, 1)	0 (0, 0)	**0.008**	**0.013**
Acanthocytes	0 (0, 1)	0 (0, 0)	**0.031**	**0.04**
Hypochromasia	0.5 (0, 1)	0 (0, 0)	**0.002**	**0.004**
Keratocytes	0 (0, 1)	0 (0, 0)	**0.03**	**0.04**
Leptocytes	0 (0, 1)	0 (0, 0)	**0.008**	**0.013**
Macrocytosis	1 (1, 1)	0 (0, 1)	**0.008**	**0.013**
Howell-Jolly bodies	1 (1, 1)	0 (0, 0)	**<0.001**	**0.002**
Schistocytes	0 (0, 1)	0 (0, 0)	**0.03**	**0.04**
**Platelets**				
Platelet concentration (×10^9^/L)	131 (49, 190)	268 (228, 299)	**<0.001**	**0.002**
Mean platelet volume (fL)	17.9 (14.2, 19.5)	10.9 (10.4, 12.1)	**<0.001**	**0.002**
Platelet volume distribution width (%)	68.4 (66.4, 70.4)	57.2 (52.2, 62.3)	**<0.001**	**0.002**
Plateletcrit (%)	0.19 (0.09, 0.27)	0.3 (0.25, 0.37)	**0.008**	**0.013**
Mean platelet component (g/dL)	21.0 (20.2, 22.1)	21.1 (20.1, 21.6)	0.89	0.89
Platelet component distribution width (g/dL)	6.5 (6, 6.8)	6 (4.4, 6.8)	0.20	0.22
Mean platelet mass (pg)	2.89 (2.46, 3.07)	2.12 (2.03, 2.31)	**<0.001**	**0.002**
Platelet dry mass distribution width (pg)	1.29 (1.23, 1.46)	0.8 (0.76, 0.87)	**<0.001**	**0.002**
Platelet Aggregation	0 (0, 3)	1 (0, 2)	0.38	0.59
**Platelet morphology**				
Giant Platelets	1 (1, 1)	0 (0, 1)	**0.02**	**0.03**
**Acute phase proteins**				
C-reactive protein (mg/L)	46.2 (35.0, 64.0)	<10 (<10, <10)	**<0.001**	**0.002**
Serum amyloid A (mg/L)	31.4 (20.6, 46.0)	<2 (<2, <2)	**<0.001**	**0.002**

* Presented as median (interquartile range). ^†^ Based on Mann–Whitney U tests, with significance set as *p* < 0.05. The *p*-value for morphology based on Fisher–Freeman–Halton exact test; the *p*-value for echinocytes was not significant, and a *p*-value could not be calculated for the following morphological variables as the median and IQRs were zero: spherocytes, microcytosis and poikilocytosis. BH: Benjamini–Hochberg.

**Table 4:** Erythrocyte and platelet characteristics of tumor-bearing dogs without intracavitary hemorrhage with sarcoma compared to tumor-bearing dogs with carcinoma.

Variable	Dogs with Carcinoma * (*n* = 28)	Dogs with Sarcoma * (*n* = 21)	*p* Value ^†^
**Complete blood count**			
**Erythrocytes**			
Hemoglobin (g/L)	12.8 (9.90, 15.2)	14.3 (9.90, 14.8)	0.86
Red blood cell count (×10^12^/L)	5.50 (4.70, 6.77)	6.07 (4.60, 6.40)	0.89
Hematocrit (%)	0.39 (0.31, 0.45)	0.43 (0.30, 0.44)	0.88
Mean cell volume (fL)	67.9 (66.8, 70.1)	67.6 (65.5, 70.2)	0.59
Mean cell hemoglobin concentration (g/dL)	32.3 (32.3, 33.8)	32.8 (32.6, 33.4)	0.27
Red cell distribution width (fL)	14.0 (13.3, 15.6)	13.7 (13.0, 15.2)	0.40
Reticulocyte percentage (%)	1.80 (1.15, 2.30)	1.90 (1.20, 3.95)	0.65
Absolute reticulocyte count (×10^9^/L)	89.2 (70.8, 140)	102 (65.3, 193)	0.83
Reticulocyte hemoglobin content (pg/cell)	24.9 (22.9, 26.0)	24.4 (23.6, 25.1)	0.93
Reticulocyte mean cell volume (fL)	88.4 (83.7, 92.2)	85.0 (82.1, 89.0)	0.07
**Platelets**			
Platelet concentration (×10^9^/L)	416 (282, 630)	355 (289, 471)	0.41
Mean platelet volume (fL)	12.5 (10.5, 13.4)	11.8 (10.8, 12.9)	0.63
Platelet volume distribution width (%)	66.3 (61.4, 70.0)	62.1 (60.3, 66.0)	0.07
Plateletcrit (%)	0.54 (0.37, 0.64)	0.41 (0.32, 0.52)	0.19
Mean platelet component (g/dL)	21.0 (20.1, 22.3)	21.2 (18.9, 22.4)	0.87
Platelet component distribution width (g/dL)	6.7 (5.8, 6.7)	6.3 (5.4, 7.0)	0.45
Mean platelet mass (pg)	2.13 (1.97, 2.32)	2.06 (1.94, 2.39)	0.61
Platelet dry mass distribution width (pg)	0.88 (0.78, 1.08)	0.86 (0.80, 0.98)	0.98
Platelet Aggregation	0 (0, 1)	1 (0, 1)	0.704
**Acute phase proteins**			
C-reactive protein (mg/L)	51.5 (32.4, 107)	97.5 (52.5, 197)	0.12
Serum amyloid A (mg/L)	22.7 (6.21, 101)	49.3 (11.4, 206)	0.45

* Presented as median (interquartile range). ^†^ Based on Mann–Whitney U tests, with significance set as *p* < 0.05. Where calculated, the *p*-value for all erythrocyte and platelet morphology was not significant. A *p*-value could not be calculated for the following morphological variables as the median and IQRs were zero: echinocytes, spherocytes, hypochromasia, keratocytes, leptocytes, microcytosis, schistocytes and poikilocytosis. Correlation is seen as significant at the 0.05 level (2-tailed).

**Table 5:** Erythrocyte and platelet variables of tumor-bearing dogs with metastasis compared to the variables of tumor-bearing dogs without metastasis, as well as correlations of CRP and SAA to erythrocyte and platelet variables.

Variable	Tumor-Bearing Dogs with Metastasis * (*n* = 25)	Tumor-Bearing Dogs Without Metastasis * (*n* = 24)	*p* Value ^†^	BH Corrected *p* Value	CRP r_s_ to Variable, with Metastasis (*p* Value; BH Corrected *p* Value)	SAA r_s_ to Variable, with Metastasis (*p* Value; BH Corrected *p* Value)	CRP r_s_ to Variable, Without Metastasis (*p* Value; BH Corrected *p* Value)	SAA r_s_ to Variable, Without Metastasis (*p* Value; BH Corrected *p* Value)
**Complete blood count**								
**Erythrocytes**								
Hemoglobin (g/L)	127 (99, 157)	132 (105, 146)	0.73	0.84	−0.46 (**0.020**; 0.11)	−0.443 (**0.027**; 0.22)	−0.35 (0.09; 0.38)	−0.47 (**0.021**; 0.18)
Red blood cell count (×10^12^/L)	5.87 (4.74, 6.95)	5.51 (4.57, 6.37)	0.50	0.84	−0.43 (**0.032**; 0.11)	−0.36 (0.08; 0.22)	−0.29 (0.17; 0.47)	0.42 (**0.039**; 0.16)
Hematocrit (%)	0.4 (0.3, 0.46)	0.39 (0.32, 0.43)	0.76	0.84	−0.47 (**0.019**; 0.11)	−0.39 (0.052; 0.22)	−0.32 (0.13; 0.44)	−0.45 (**0.028**; 0.16)
Mean cell volume (fL)	66.8 (63.8, 68.1)	68.4 (67, 70.5)	**0.005**	0.08	−0.13 (0.53; 0.76)	−0.20 (0.34; 0.45)	−0.03 (0.90; 0.96)	0.06 (0.76; 0.92)
Mean cell hemoglobin concentration (g/dL)	33 (32.4, 33.7)	32.9 (32.5, 33.5)	0.72	0.84	-	-	-	-
Red cell distribution width (fL)	14.2 (13.3, 15.7)	13.5 (12.7, 15.2)	0.17	0.84	0.28 (0.18; 0.27)	0.36 (0.07; 0.22)	0.17 (0.42; 0.79)	0.26 (0.22; 0.41)
Reticulocyte percentage (%)	2 (1.4, 3.6)	1.8 (1.1, 2.5)	0.26	0.84	0.43 (**0.033**; 0.11)	0.44 (**0.026**; 0.22)	0.08 (0.70; 1.0)	0.21 (0.33; 0.56)
Absolute reticulocyte count (×10^9^/L)	121 (70.4, 193)	87.2 (65.2, 110)	0.11	0.77	0.280 (0.17; 0.27)	0.33 (0.11; 0.22)	−0.08 (0.69; 0.79)	0.02 (0.92; 0.92)
Reticulocyte hemoglobin content (pg/cell)	25 (23.1, 25.4)	24.3 (23.2, 25.6)	0.66	0.84	−0.38 (0.06; 0.15)	−0.33 (0.11; 0.22)	−0.59 (**0.003**, 0.051)	−0.70 **(<0.001; 0.017)**
Reticulocyte mean cell volume (fL)	87.3 (83.6, 89.8)	87 (81.4, 91)	0.72	0.84	−0.07 (0.74; 0.85)	−0.02 (0.91; 0.96)	−0.43 (0.05; 0.43)	−0.44 (**0.047**; 0.16)
**Platelets**								
Platelet concentration (×10^9^/L)	382 (302, 586)	404 (255, 589)	0.71	0.84	−0.02 (0.92; 0.92)	−0.04 (0.84; 0.95)	0.2 (0.36; 0.76)	0.28 (0.18; 0.38)
Mean platelet volume (fL)	12.3 (10.5, 13.9)	12.1 (10.7, 12.9)	0.67	0.84	0.42 (**0.038**; 0.11)	0.32 (0.12; 0.22)	0.11 (0.61; 0.79)	0.04 (0.83; 0.92)
Platelet volume distribution width (%)	63.3 (60.4, 70.9)	64.5 (61.8, 67.5)	0.91	0.91	0.33 (0.11; 0.23)	0.22 (0.30; 0.45)	0.14 (0.51; 0.79)	−0.02 (0.92; 0.92)
Plateletcrit (%)	0.48 (0.36, 0.61)	0.43 (0.32, 0.59)	0.55	0.84	0.07 (0.75; 0.85)	−0.01 (0.96; 0.96)	0.24 (0.25; 0.61)	0.36 (0.08; 0.23)
Mean platelet component (g/dL)	21.2 (19.9, 22.4)	20.8 (19.9, 22.2)	0.72	0.84	−0.06 (0.77; 0.85)	−0.20 (0.33; 0.45)	−0160 (0.46; 0.79)	−0.16 (0.44; 0.68)
Platelet component distribution width (g/dL)	6.5 (5.9, 7.4)	6.6 (5.5, 7.1)	0.88	0.91	0.05 (0.80; 0.85	0.14 (0.506; 0.61)	−0.4 (0.05; 0.30)	−0.33 (0.12; 0.28)
Mean platelet mass (pg)	2.15 (1.97, 2.35)	2.12 (1.96, 2.31)	0.60	0.84	0.29 (0.16; 0.27)	0.21 (0.31; 0.45)	0.01 (0.96; 0.96)	−0.05 (0.81; 0.92)
Platelet dry mass distribution width (pg)	0.9 (0.8, 1.07)	0.88 (0.76, 0.95)	0.34	0.84	0.48 (**0.014**, 0.11)	0.41 (**0.040**; 0.22)	0.14 (0.51; 0.72)	0.05 (0.80; 0.92)
**Platelet morphology**								
Giant Platelets	0 (0, 1)	1 (1, 1)	**0.008**	0.08	-	-	-	-
**Acute phase proteins**								
C-reactive protein (mg/L)	78.7 (42.8, 160)	58.0 (31.6, 111)	0.64	0.84	-	-	-	-
Serum amyloid A (mg/L)	38.9 (11.4, 122)	22.6 (9.17, 161)	0.62	0.84	-	-	-	-

* Presented as median (interquartile range). Spearman’s coefficient is denoted by r_s._ ^†^ Based on Mann–Whitney U tests, with significance set as *p* < 0.05. The *p*-value for morphology based on Fisher–Freeman–Halton exact test. Where calculated, the *p*-value for all erythrocyte morphology was not significant. A *p*-value could not be calculated for the following morphological variables as the median and IQRs were zero: echinocytes, spherocytes, hypochromasia, keratocytes, leptocytes, microcytosis, schistocytes and poikilocytosis. Correlation is seen as significant at the 0.05 level (2-tailed). BH: Benjamini–Hochberg.

## Data Availability

The original contributions presented in this study are included in the article. Further inquiries can be directed to the corresponding author(s).

## Abbreviations

ARC	Absolute reticulocyte count
CBC	Complete blood count
CHr	Reticulocyte hemoglobin content
CRP	C-reactive protein
HCT	Hematocrit
HGB	Hemoglobin
IL-1	Interleukin 1
IL-1α	Interleukin 1 alpha
IL-2	Interleukin 2
IL-6	Interleukin 6
MCHC	Mean cell hemoglobin concentration
MCV	Mean cell volume
MCVr	Reticulocyte mean corpuscular volume
MPC	Mean platelet component concentration
MPM	Mean platelet mass
MPV	Mean platelet volume
PCT	Plateletcrit
PCDW	Platelet component distribution width
PDW	Platelet volume distribution width
PLT	Platelet concentration
PMDW	Platelet dry mass distribution width
PTHR1	Parathyroid hormone receptor 1
RBC	Red blood cell count/concentration
RET%	Reticulocyte percentage
RDW	Erythrocyte distribution width
SAA	Serum amyloid A
TNF-α	Tumor necrosis factor alpha

## Appendix A

**Table A1:** Signalment of tumor-bearing dogs and the healthy control population.

	Median Age in Years (IQR)	Sex	Breeds
Tumor-bearing Dogs	11 (9.0–13.6)	FI: 20 FS: 16 MI: 9 MN:14	Mixed breed (8), German shepherd dog (7), Jack Russel terrier (7), Boerboel (6), dachshund (5), Labrador retriever (5), cocker spaniel (2), fox terrier (2), American pitbull terrier (2), pug (2), Staffordshire bull terrier (2), and one each of beagle, border collie, bullmastiff, rottweiler, Scottish terrier, bull terrier, Pekingese, Pomeranian, standard poodle, Great Dane, Irish terrier
Healthy unaffected dogs	10.25 years (9.3–11.9)	FS: 9 MN: 9 MI: 2	Mixed breed (8), Jack Russel terrier (4), dachshund (2) and one each of basset hound, German shepherd dog, bull terrier, Staffordshire bull terrier, miniature French poodle, Pekingese

Abbreviations: FI, female intact; FS, femal sterilized; IQR: interquartile range; MI, male intact; MN, male neutered.

**Table A2:** Primary carcinoma or sarcoma with concurrent secondary or tertiary tumors, or concurrent inflammation, was identified in the dogs included in the study. Each primary tumor listed was associated with one individual dog in the study population.

Primary Tumor	Secondary Tumor	Tertiary/Quaternary Tumor or Concurrent Inflammation Identified
**Sarcomas**		
Subcutaneous soft tissue sarcoma	Focal hepatic cystadenoma	
Splenic hemangiosarcoma	Round cell tumor of the face	Multifocal, moderate, acute to subacute renal tubular and medullary necrosis
Right atrial hemangiosarcoma	Focal hepatocellular adenoma	Focal adrenal cortical nodular hyperplasia
Splenic & right atrial hemangiosarcoma	Pheochromocytoma	
Hepatic hemangiosarcoma	Adrenal cortical carcinoma	
Hepatic hemangiosarcoma		Focal chronic-active pyogranulomatous aortic lesion with intralesional *Spirocerca lupi*
Cutaneous hemangiosarcoma	Pulmonary bronchoalveolar carcinoma	Pheochromocytoma Moderate chronic lympho-histiocytic interstitial nephritis Bacterial cystitis
Cutaneous hemangiosarcoma	Early splenic stromal sarcoma	Renal adenomas Mild granulomatous cholangiohepatitis
Subcutaneous hemangiosarcoma	Thyroid follicular carcinoma	
Mammary osteosarcoma	Mammary mixed-type carcinoma	Multifocal adrenal cortical adenomas
Mammary osteosarcoma		Bacterial cystitis
Maxillary osteosarcoma	Compact thyroid carcinoma of the ascending aorta	
Mandibular osteosarcoma	Pulmonary squamous cell carcinoma (primary organ not identified)	
Splenic stromal sarcoma	Simple mammary intraductal papillary carcinoma	
Muscle soft tissue sarcoma	Subcutaneous hemangioma	Bacterial prostatic abscess & cystitis
Cutaneous soft tissue sarcoma	Suspected indolent lymphoma in part of the duodenal lymph node	
**Carcinomas**		
Complex mammary carcinoma	Simple tubulopapillary mammary carcinoma	Bronchoalveolar adenoma Adrenal cortical adenoma
Complex mammary carcinoma	Metastatic melanoma (metastasis to inguinal ln and liver), source unknown	Hepatocellular adenoma
Complex mammary carcinoma	Oesophageal osteosarcoma (*S. lupi*)	Bacterial cystitis
Simple tubular mammary carcinoma	Subcutaneous soft tissue sarcoma (hindlimb)	
		
Simple tubular mammary carcinoma	Complex mammary carcinoma	Subcutaneous mast cell tumor Subcutaneous hemangioma Benign fibrolipoma Bacterial cystitis
Mixed mammary carcinoma	Compact thyroid carcinoma	Adrenal cortical carcinoma
Mixed mammary carcinoma	Cutaneous melanoma (metastatic to inguinal lymph node)	Bacterial cystitis
Anaplastic mammary carcinoma	Complex mammary carcinoma	
Simple tubulopapillary mammary carcinoma	Focal scirrhous pulmonary carcinoma	Severe chronic, multifocal to extensive, renal interstitial fibrosis & lymphoplasmacytic nephritis Bacterial cystitis
Simple tubular mammary carcinoma	Benign oesophageal *Spirocerca lupi* nodule	
Mixed-type carcinoma & simple tubulopapillary mammary carcinoma		*Spirocerca lupi* migratory track: severe mural inflammation of the aorta
Mammary ductal carcinoma	Pheochromocytoma	Mild chronic interstitial lymphocytic and fibrosing nephritis
Sinonasal transitional carcinoma	Benign oesophageal *Spirocerca lupi* nodule	
Squamous cell carcinoma		Multifocal granulomatous nephritis
Squamous cell carcinoma		Chronic interstitial nephritis and a chronic membranous glomerulopathy
Solid scirrhous prostatic carcinoma		Focal, subacute, mild aspiration pneumonia
